# Lung isolation—a personalized and clinically adapted approach to control bronchoscopy-associated acute massive airway hemorrhage

**DOI:** 10.1186/s12890-023-02780-2

**Published:** 2023-11-30

**Authors:** Mingyuan Yang, Yunzhi Zhou, Hong Li, Huafeng Wei, Qinghao Cheng

**Affiliations:** 1 Center of Anesthesiology and Pain, Emergency General Hospital, Beijing, 100028 China; 2 Department of Pulmonary and Critical Care Medicine, Emergency General Hospital, Beijing, China; 3 Department of Anesthesiology and Critical Care, University of Pennsylvania, Philadelphia, PA 19104 USA

**Keywords:** Bronchoscopy-associated acute massive airway hemorrhage, Bleeding volume, Bleeding rate, Impact on pulmonary oxygenation function, Hemostatic methods, Lung isolation, Mortality

## Abstract

**Background:**

The current concept of bronchoscopy-associated massive airway hemorrhage is not accurate enough, and the amount of bleeding as the only evaluation criterion cannot comprehensively evaluate magnitude of the effects and the severity.

**Objective:**

To propose the concept of bronchoscopy-associated acute massive airway hemorrhage, analyze its impact on patients and highlight the treatment approach of acute massive airway hemorrhage without ECMO support.

**Designs:**

A retrospective cohort study.

**Setting:**

Include all patients who received bronchoscopy intervention therapy at Interventional Pulmonology Center of Emergency General Hospital from 2004 to December 2021.

**Patients:**

223 patients met the inclusion criteria.

**Intervention:**

Patients were divided into two groups: acute massive airway hemorrhage group (n = 29) and non-acute massive airway hemorrhage group (n = 194).

**Main outcome measures:**

Perioperative adverse events between two groups were the main outcome. Secondary outcome was the impact of lung isolation on patient in group Acute.

**Results:**

The incidence of acute massive airway hemorrhage was 0.11%, and the incidence of non-acute massive airway hemorrhage was 0.76% in this study. There were significant differences in the incidence of intraoperative hypoxemia, lowest SpO_2_, hemorrhagic shock, cardiopulmonary resuscitation, intraoperative mortality, and transfer to ICU between acute group and non-acute group (*P*<0.05, respectively). Lung isolation was used in 12 patients with acute massive airway hemorrhage, and only 2 patients died during the operation.

**Conclusion:**

Bronchoscopy-associated acute massive airway hemorrhage had more serious impact on patients due to rapid bleeding, blurred vision of bronchoscopy, inability to stop bleeding quickly, blood filling alveoli, and serious impact on oxygenation of the lung lobes. Polyvinyl chloride single-lumen endotracheal intubation for lung isolation, with its characteristics of low difficulty, wide applicability and available in most hospitals, may reduce the intraoperative mortality of patients with bronchoscopy-associated acute massive airway hemorrhage.

**Trial registration:**

Chinese Clinical Trial Registry on 13/03/2022. Registration number: ChiCTR2200057470.

## Key Points

Bronchoscopy-associated acute massive airway hemorrhage was considered more accurate and meaningful to assess the magnitude of the effects and the severity according to the bleeding volume, bleeding rate, and impact on pulmonary oxygenation function.

It was defined as massive hemorrhage of more than 100 ml caused by bronchoscopy, rapid outflow of blood from the bleeding point, blurred vision of bronchoscopy, inability to stop bleeding quickly, and blood filling the alveoli, which seriously affects the oxygen supply function of the lung lobes.

Compared with non-acute massive airway hemorrhage, acute massive airway hemorrhage had significant differences in blood loss volume, duration and difficulty of therapy, hemostatic methods and perioperative complications, which brings different effects to patients.

Once acute massive airway hemorrhage occurs, it was an emergency, and an effective, feasible and adaptable hemostatic measure was needed to meet the dual functions of ventilation and hemostasis, so as to win valuable time and opportunity for follow-up treatment.

Polyvinyl chloride single-lumen endotracheal intubation for lung isolation, with its characteristics of low difficulty, wide applicability and available in most hospitals, may reduce the intraoperative mortality of patients with bronchoscopy-associated acute massive airway hemorrhage.

## Background

Bronchoscopy intervention developed rapidly and had been widely used for both initial diagnosis and follow-up treatment of severe respiratory diseases. Subsequently, it was more and more applied to improve the ventilation problems of patients with benign and malignant central airway obstruction [1]. Massive hemorrhage of lower respiratory tract with a single blood loss of > 100 mL caused by bronchoscopy diagnosis and treatment is called bronchoscope-associated massive airway hemorrhage [2, 3]. The incidence and mortality of bronchoscope-associated massive airway hemorrhage are exceedingly rare [4, 5], but once it occurs, the incidence of intraoperative adverse events and mortality are relatively high, that are predominantly induced by therapeutic rather than diagnostic bronchoscopies, such as bronchoscopy biopsy and tumor resection [6]. Studies from a decade ago showed a incidence of 1.56‰ rate from 1998 to 2004 [7] and a incidence of 8.5‰ from 2004 to 2011in China [8].

Massive airway hemorrhage is the most serious complication of bronchoscopy intervention therapy. It can produce a number of serious consequences compared with other surgical hemorrhage, causing severe airway obstruction and a severe reduction in pulmonary oxygenation with minimal loss of blood volume [9]. The effects on respiratory oxygenation appear earlier, faster, more urgent and more dangerous than the circulatory change based on blood volume, which will in turn aggravate the effects on circulation, leading to problems such as hypoxia-induced cardiac arrest.

The concept of bronchoscopy-associated acute massive airway hemorrhage had not been proposed, and existing literatures mainly focused on the amount of intraoperative blood loss [10, 11]. However, it was not accurate to use blood loss volume alone to assess the severity of bronchoscopy-associated massive airway hemorrhage. It was considered more accurate and meaningful to assess the magnitude of the effects and the severity of bronchoscopy-associated massive airway hemorrhage according to the bleeding volume, bleeding rate, and impact on pulmonary oxygenation function.

Veno-venous extracorporeal membrane oxygenation (VV-ECMO) provided a safer environment for patients undergoing bronchoscopy interventional procedures for severe airway problems, such as high risk of airway obstruction and endobronchial bleeding [12]. While, as the most advanced life support tool, extracorporeal membrane oxygenation (ECMO) had been used in a relatively small and disproportionate proportion in non-general hospitals under the background of large population base, relatively tight medical resources and rapid development of airway interventional therapy.

Bronchoscope-associated acute massive airway hemorrhage was considered to have more serious impact on patients due to the rapid and large amount of bleeding, which may cause more serious adverse reactions and require different rescue methods. The purpose of this study was to propose the concept of bronchoscopy-associated acute massive airway hemorrhage, analyze its impact on patients through comprehensive evaluation with bronchoscopy-associated non-acute massive airway hemorrhage, and highlight the treatment approach of acute massive airway hemorrhage without ECMO support.

## Methods

### Study design and patients

#### Ethics

This was a retrospective cohort study that included all patients who received bronchoscopy intervention therapy at the tertiary Interventional Pulmonology Center of Emergency General Hospital from 2004 to December 2021. Ethical approval for this study (Ethical Committee Number: K21-39) was provided by the Ethical Committee of Emergency General Hospital, Beijing, China (Chairperson Prof Qingyu Zeng) on 29 November 2021. It was registered in the Chinese Clinical Trial Registry on 13/03/2022 (Registration number: ChiCTR2200057470). All patients of Interventional Pulmonology Center were informed that the patients’ clinical data might be used in clinical studies and signed the informed consent form on admission and before bronchoscopy intervention therapy.

#### Patient and public involvement

Patients or the public were not involved in the design, or conduct, or reporting, or dissemination plans of our research.

#### Study population

Inclusion criteria: [1] Diagnosed of airway lesions; [2] Scheduled for bronchoscopy intervention therapy; [3] Intraoperative blood loss was more than 100ml. Exclusion criteria: Patients who refuse to use their medical records for clinical research and medical studies.

Patients were assigned into two groups according to intraoperative bleeding volume, bleeding rate, and impact on pulmonary oxygenation function. Patients with blood loss of more than 100ml, rapid bleeding leading to blurred vision under bronchoscopy and difficulty in hemostasis, and a significant short-term plummeting in pulse oxygen saturation (SpO_2_) (less than 80%) were enrolled in the bronchoscopy-associated acute massive airway hemorrhage group (Group Acute). Patients with blood loss of more than 100ml, cleared vision bronchoscopy and easy in hemostasis, and maintained SpO_2_ (above than 80%) were enrolled in the bronchoscopy-associated non-acute massive airway hemorrhage group (Group Non-acute).

**Fig. 1:**
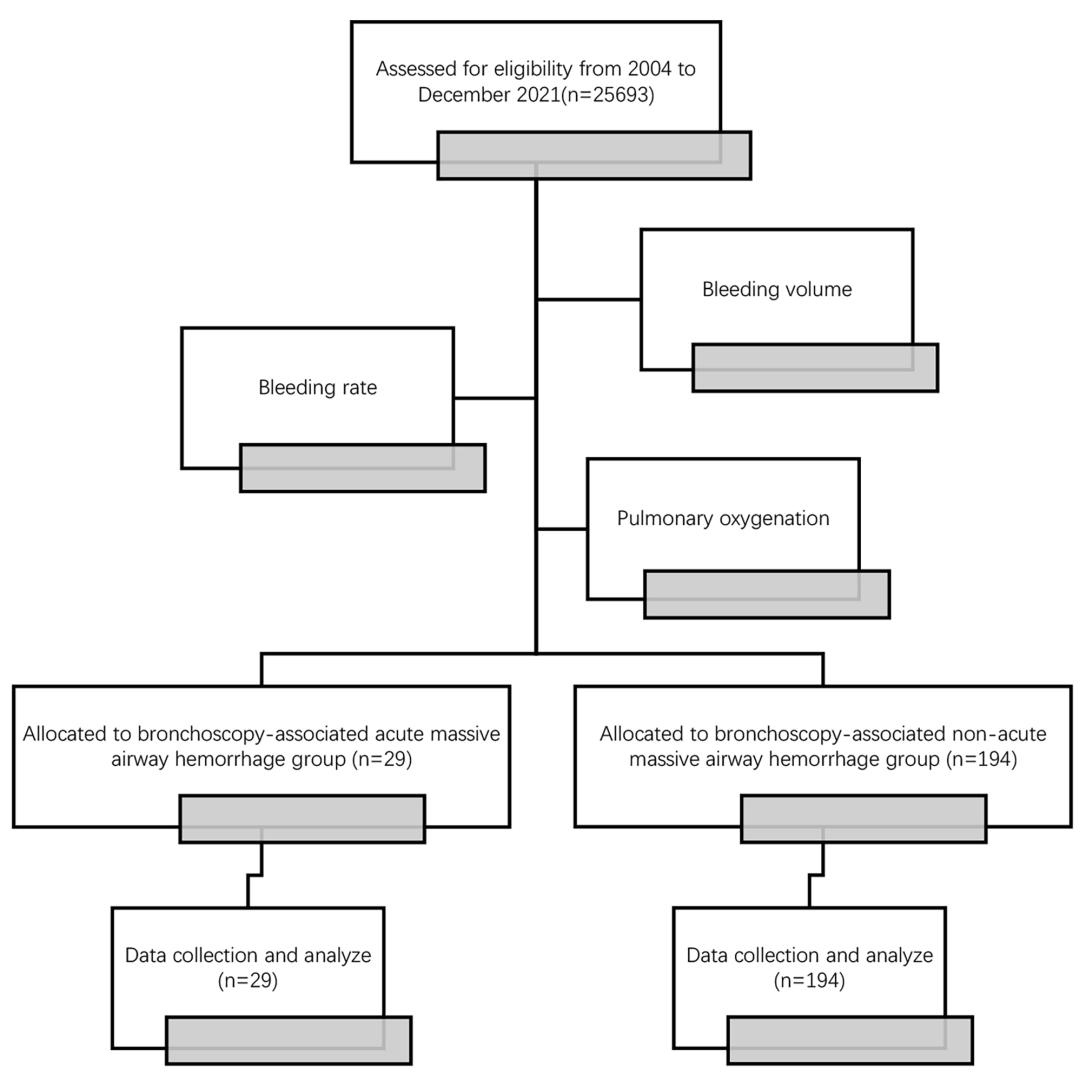
Flow Diagram

The flow diagram of this study is shown in Fig. 1.

#### Data collection

Collected data were mainly divided into three parts: pre-operative characteristics, intra-operative bronchoscope intervention characteristics and perioperative adverse events.

Pre-operative characteristics were consisted of demographic and clinical data, including age, gender, lesion etiology, Karnofsky Performance Status (KPS) score [13], modified British medical research council (MMRC) score [14], preoperative degree of stenosis, proportion of urgent cases, and incidence of preoperative bronchial artery embolization (BAE). Lesion etiology includes lung cancer, malignant metastatic tumor and benign lesion.

**Fig. 2:**
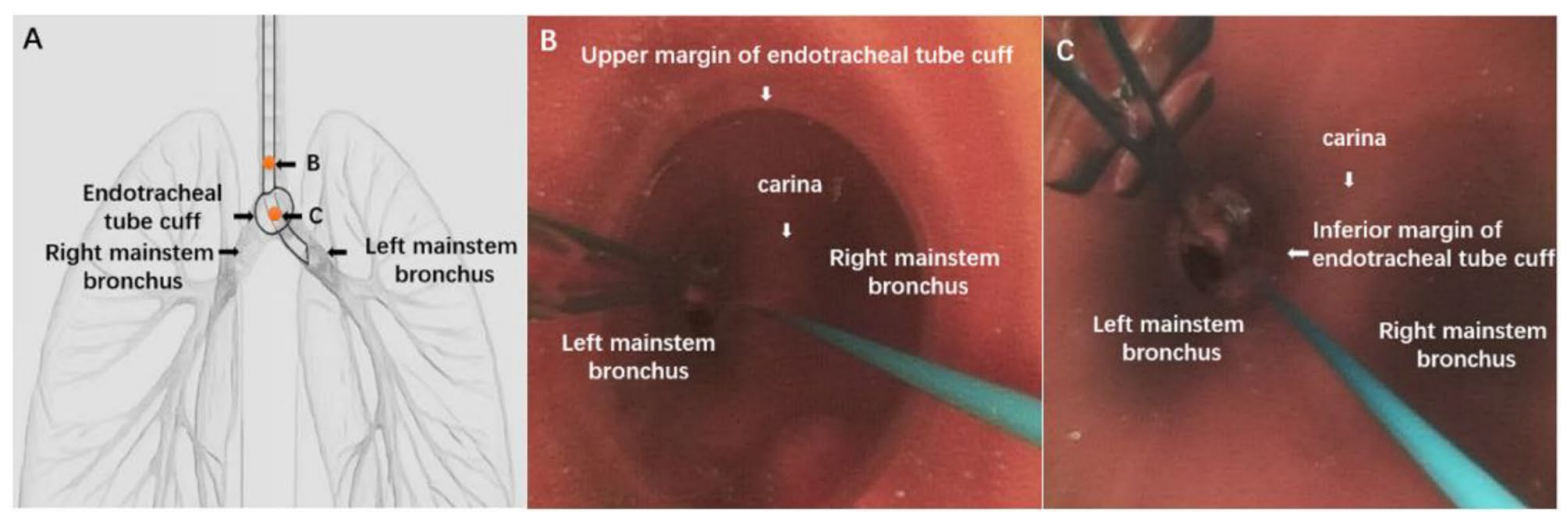
Positioning Schematic Diagram of Lung Isolation by Endotracheal tube. (**A**) The tip of the endotracheal tube was inserted into the mainstem bronchus on the un-bleeding side, and the cuff is fixed on the carina to prevent the blood on the bleeding side from overflowing. (**B**) The flexible electronic bronchoscope showed the appearance of left mainstem bronchus, right mainstem bronchus, carina and the upper margin of the cuff at the position of point B in the endotracheal tube. (**C**) The flexible electronic bronchoscope showed the appearance of left mainstem bronchus, right mainstem bronchus, carina and the inferior margin of the cuff at the position of point C in the endotracheal tube

Intra-operative bronchoscopy intervention therapy characteristics included hemorrhage location, cause of hemorrhage, conventional hemostasis method, lung isolation and compression hemostasis, intravenous infusion of hemostatic, blood loss volume, and duration of bronchoscopy intervention therapy. Hemorrhage location was divided into three locations. Location I: hemorrhage lesions were located in the main airway; Location II: hemorrhage lesions were located in the left/right mainstem bronchus; Location III: hemorrhage lesions were located both in the main airway and mainstem bronchus. Cause of hemorrhage included tumor excision hemorrhage, biopsy hemorrhage, stent extraction and implantation hemorrhage, dilation and tearing hemorrhage. Conventional basic hemostasis meant that the hemostatic effect could be achieved by bronchoscopy lavage of ice saline (4 °C), hemostatic drugs (hemocoagulase), vasoactive drugs (diluted (1:10000) adrenalin), and bronchoscopy thermal coagulation, including electroacupuncture, laser and argon plasma coagulation (APC) in the clear field of vision under electronic flexible bronchoscope. Lung isolation approach included endotracheal tube isolation and endoscopic balloon isolation. When endotracheal tube was used for lung isolation, its position was shown in Fig. 2. A disposable, quantifiable negative pressure suction device was utilized in each patient during bronchoscopy interventional therapy. Intraoperative blood loss was defined as the volume of the suction device at the end of surgery minus the amount of normal saline used during the procedure.

Perioperative adverse events included incidence of intraoperative hypoxemia (SpO_2_<90%), lowest SpO_2_, hemorrhagic shock, cardiopulmonary resuscitation, intraoperative mortality, and transfer to intensive care unit (ICU) with intubation after operation.

The primary outcomes of the study were defined as the differences in terms of bleeding volume, hemostasis method, and impact on oxygenation between group Acute and Non-acute. The secondary outcome was defined to assess the impact of lung isolation on mortality in the acute massive airway hemorrhage group.

### Statistical analysis

SPSS was used for data statistical analysis. In the collected patient data, measurement data was presented in the form of mean ± standard deviation, and counting data was presented in terms of quantity and percentage. Statistical counting data between two groups were compared by chi-square test. The measurement data between groups were compared by Univariate analysis of variance. When *P*<0.05, the data was considered statistically different.

## Results

From 2004 to December 2021, a total of 25,693 patients underwent bronchoscopy intervention therapy under anesthesia in our hospital. According to the inclusion criteria, a total of 223 patients met the criteria for massive airway hemorrhage and were assigned to two groups based on intraoperative bleeding volume, bleeding rate, and impact on pulmonary oxygenation function: acute group (n = 29) and non-acute group (n = 194). The overall incidence of airway massive hemorrhage was 0.87%, with acute and non-acute incidences at 0.1% and 0.76%, respectively.

Comparison of pre-operative characteristics between acute group and non-acute group were shown in Table 1. There was no significant difference in age, gender and lesion etiology between acute group and non-acute group. While preoperative KPS score of patients in acute group was significantly lower than that of patients in non-acute group (*P* = 0.037); and preoperative MMRC score of patients in acute group was significantly higher than that of patients in non-acute group (*P* = 0.004). Preoperative stenosis degree of patients in acute group was more severe than that in non-acute group (*P* = 0.004). Proportion of urgent cases for emergency operation and incidence of preoperative BAE in acute group were significantly higher than those in non- acute group (*P* = 0.006, *P* = 0.017). The details were shown in Table 1.

**Table 1:** Comparison of pre-operative characteristics between group Acute and Non-acute

Group	Group Acute (n = 29)	Group Non-acute (n = 194)	*P* value
**Age, years**	59.79 ± 12.86	56.38 ± 15.06	0.248
**Male, n (%)**	21(72.4)	135(69.6)	0.831
**Lesion etiology**			0.258
Lung cancer, n (%)	18(62.1)	157(75.8)	
Malignant metastatic tumor, n (%)	3(10.3)	10(5.2)	
Benign lesion, n (%)	8(27.6)	39(19.1)	
**Preoperative KPS score**	65.86 ± 12.68^*^	73.62 ± 13.29	**0.037**
**Preoperative MMRC score**	2.66 ± 1.08^*^	2.21 ± 1.08	**0.004**
**Preoperative degree of stenosis, %**	75.34 ± 21.59^*^	61.98 ± 23.54	**0.004**
**Proportion of urgent cases, n (%)**	4(13.8) ^*^	3(1.5)	**0.006**
**Bronchial artery embolization, n (%)**	3(10.3) ^*^	2(1.0)	**0.017**

Data were expressed as mean ± standard deviation or as numbers and percentages. ^*^ was statistically significant compared with Group Non-acute

KPS score: Karnofsky Performance Status score; MMRC score: modified British medical research council score

Group Acute: bronchoscope-related acute massive airway hemorrhage group; Group Non-acute: bronchoscope-related non-acute massive airway hemorrhage group

There was no significant difference in hemorrhage location and cause of hemorrhage between acute group and non-acute group. Nevertheless, conventional hemostasis method, lung isolation and compression hemostasis method, intravenous infusion of hemostatic, blood loss volume and duration of bronchoscopy intervention therapy were all significantly different between acute group and non-acute group (*P*<0.005, respectively). The utilization rate of conventional hemostasis methods in acute group was significantly lower than that in non-acute group (89.7% vs. 100%). Patients in the acute group were more likely than those in the non-acute group to use lung isolation, including endotracheal tube isolation (utilization rate 21.6% vs. 1.5%) and endoscopic balloon isolation (utilization rate 23.5% vs. 1.5%), in addition to conventional hemostasis. At the same time, patients in the acute group were more likely to use intravenous hemostatic drugs (86.2% vs. 37.6%). Blood loss volume of patients in acute group was significantly more than that in non-acute group (1198.28ml vs. 216.18ml), and duration of bronchoscopy intervention therapy of patients in acute group was significantly longer than that in non-acute group (93.90 min vs. 67.80 min). The details were shown in Table 2.

**Table 2:** Comparison of intra-operative bronchoscope intervention characteristics between group Acute and Non-acute

Group	Group Acute (n = 29)	Group Non-acute (n = 194)	*P* value
**Hemorrhage location**			0.872
Location I, n (%)	4(13.8)	27(13.9)	
Location II, n (%)	23(79.3)	158(81.4)	
Location III, n (%)	2(6.9)	9(4.6)	
**Cause of Hemorrhage**			0.621
Tumor excision, n (%)	19(65.5)	127(65.5)	
Biopsy, n (%)	7(24.1)	57(29.4)	
Stent extraction and implantation, n (%)	2(6.9)	8(4.1)	
Dilation and tearing, n (%)	1(3.4)	2(1.0)	
**Conventional hemostasis method, n (%)**	26(89.7) ^*^	194(100)	**0.002**
**Lung isolation approach**			
Endotracheal tube isolation, n (%)	8(21.6) ^*^	3(1.5)	**0.000**
Endoscopic balloon isolation, n (%)	4(23.5) ^*^	3(1.5)	**0.006**
**Intravenous infusion of hemostatic, n (%)**	25(86.2) ^*^	73(37.6)	**0.000**
**Blood loss volume, ml**	1198.28 ± 913.93^*^	216.18 ± 162.16	**0.000**
**Duration of therapy, mins**	93.90 ± 53.07 ^*^	67.80 ± 28.09	**0.000**

Data were expressed as mean ± standard deviation or as numbers and percentages. ^*^ was statistically significant compared with Group Non-acute

Location I: hemorrhage lesions were located in the main airway; Location II: hemorrhage lesions were located in the left/right mainstem bronchus; Location III: hemorrhage lesions were located both in the main airway and mainstem bronchus

Group Acute: bronchoscope-related acute massive airway hemorrhage group; Group Non-acute: bronchoscope-related non-acute massive airway hemorrhage group

Significantly, perioperative adverse events of patients were significantly more common with higher incidence in the acute group compared with the patients in the non-acute group including intraoperative hypoxemia (SpO_2_<90%) (100% vs. 63.4%), lowest SpO_2_ (51.31% vs. 87.76%), incidence of hemorrhagic shock (24.1% vs. 0%), incidence of cardiopulmonary resuscitation (24.1% vs. 0%), intraoperative mortality (6.9% vs. 0%), and incidence of transfer to ICU with intubation (86.2% vs. 18.0%). The details were shown in Table 3.

**Table 3:** Comparison of perioperative adverse events between group Acute and Non-acute

Group	Group Acute (n = 29)	Group Non-acute (n = 194)	*P* value
Intraoperative hypoxemia, n (%)	29(100) ^*^	123(63.4)	**0.000**
Lowest SpO_2_	51.31 ± 28.17^*^	87.76 ± 9.46	**0.000**
Hemorrhagic shock, n (%)	7(24.1) ^*^	0(0)	**0.000**
Cardiopulmonary resuscitation, n (%)	7(24.1) ^*^	0(0)	**0.000**
Intraoperative mortality, n (%)	2(6.9) ^*^	0(0)	**0.016**
Transfer to ICU with intubation, n (%)	25(86.2) ^*^	35(18.0)	**0.000**

Data were expressed as mean ± standard deviation or as numbers and percentages. ^*^ was statistically significant compared with Group Non-acute

Group Acute: bronchoscope-related acute massive airway hemorrhage group; Group Non-acute: bronchoscope-related non-acute massive airway hemorrhage group

(Figure A). The tip of the endotracheal tube was inserted into the mainstem bronchus on the un-bleeding side, and the cuff is fixed on the carina to prevent the blood on the bleeding side from overflowing

(Figure B) The flexible electronic bronchoscope showed the appearance of left mainstem bronchus, right mainstem bronchus, carina and the upper margin of the cuff at the position of point B in the endotracheal tube

(Figure C) The flexible electronic bronchoscope showed the appearance of left mainstem bronchus, right mainstem bronchus, carina and the inferior margin of the cuff at the position of point C in the endotracheal tube

Of the 29 patients with acute massive airway hemorrhage, 12 patients underwent compression hemostasis with lung isolation. In 12 patients with lung isolation, 4 cases were treated with endoscopic balloon, and 8 patients with polyvinyl chloride (PVC) single-lumen endotracheal tube. At the same time, 7 of the 12 patients had respiratory and cardiac arrest, 2 died of hemorrhagic shock during the operation, and the remaining 5 patients returned to ICU for further treatment.

## Discussion

Bronchoscopy intervention therapy had no longer been limited to the diagnosis of diseases, but also widely used in the rescue of acute and critically ill patients. At the same time, the occurrence of bronchoscope-associated acute massive airway hemorrhage caused by biopsy and tumor resection had been reported successively [5, 15–18] and increased with the development and complexity of procedures. The definition, diagnostic criteria and incidence of massive airway hemorrhage associated with bronchoscopy intervention were slightly different in different literatures, which were mostly emphasized on the volume of intraoperative blood loss [19–21]. Research of Japan Society in 2010 defined it as the blood loss of more than 300 ml during bronchoscopy intervention therapy [4]. The interventional respiratory group of Chinese Respiratory Society defined the acute lower respiratory tract massive hemorrhage caused by bronchoscopy procedure as a single hemorrhage of more than 100 ml, which was called bronchoscope-associated massive airway hemorrhage [22].

The total amount of blood loss had always been the most important criterion for the evaluation of massive hemorrhage, but due to the particularity of bronchoscopy, this index alone was not enough to comprehensively evaluate the severity of massive airway hemorrhage. So, the concept of bronchoscopy-associated acute massive airway hemorrhage was proposed in this study. In addition to the total amount of bleeding, the concept of bronchoscopy-associated acute massive airway hemorrhage introduced two key factors in the basis of previous definition: the rate of bleeding and the effect on pulmonary oxygenation. It was defined as massive hemorrhage of more than 100 ml caused by bronchoscopy, rapid outflow of blood from the bleeding point, blurred vision of bronchoscopy, inability to stop bleeding quickly, and blood filling the alveoli, which seriously affected the oxygen supply function of the lung lobes.

As the comparative results of this study showed that there were significant differences in many factors of preoperative, intraoperative and postoperative characteristics of patients between the two groups. The symptoms of breath-holding in patients and the degree of airway stenosis were more severe in acute group than those in the non-acute group. The more severe degree of stenosis and breathlessness necessitated an increase in the probability of emergency surgery, but the probability of intraoperative massive bleeding due to the nature of the lesions is still high, despite the fact that bronchial artery embolization had been performed for non-emergency patients.

There were significant differences in the total amount of blood loss, hemostatic methods, operation duration and perioperative complications between the patients with acute massive airway hemorrhage and non-acute massive airway hemorrhage. Among them, the intraoperative mortality was of great concern, which was greatly affected by the following rescue methods, had a great impact on patients and was also a great test for doctors. Compared with the non-acute group of patients, the amount of blood loss in the acute group of patients was significantly increased, and the duration of operation was prolonged. Hemostatic methods vary with the amount and rate of bleeding, and are more cumbersome and complex in acute massive airway hemorrhage. In many cases, the bleeding rate was too fast to follow the conventional hemostasis steps step by step, so it was necessary to directly take the method of lung isolation, which played a decisive role in the prognosis of patients.

Many studies had reported the benefits of VV-ECMO combined with bronchoscopic airway intervention for critically ill patients with airway problems [23, 24], but not in all patients. Although the advanced technology is good, it is not omnipotent for emergencies, and the establishment of ECMO is time-consuming and labor-intensive, requiring a long time and a lot of preparation [12], which cannot meet the principle of rapid and accurate handling of emergencies. An effective, feasible and adaptable hemostasis measure is needed to meet the dual functions of ventilation and hemostasis when acute massive airway hemorrhage breaks out during bronchoscopy airway intervention, to gain valuable time and opportunity for subsequent treatment. Lung isolation can effectively control the hemorrhage of the bleeding side and ensure the oxygenation of the patient by using the non-bleeding side lung. The two patients who died during the operation were intubated after cardiac and respiratory arrest. At that time, the patients had missed the best time for lung isolation to stop bleeding, and eventually died during the operation due to circulatory failure.

To achieve lung isolation in acute massive airway hemorrhage, bronchial blockers, endoscopic balloon and PVC endotracheal tube may be used [25, 26]. Clinical experience confirmed that use of a double-lumen endotracheal tube (DLT) was identified as the first to be avoided in the management of acute massive airway hemorrhage. Blurred vision in airway made it difficult to accurately locate the DLT in a very short time. In addition, accurate positioning of DLT need to be completed by the assistance of fiberoptic bronchoscope with an outer diameter of about 2.8 to 3.5 mm, which is easily blocked by blood clots and completely unable to check and locate DLT. In the process, DLT is not applicable and practical, not only failed to solve the problem in the tense and precious golden time, but may even exacerbate the problem, leading to further decline of SpO_2_ and even death, which should be avoided in the management of acute massive airway hemorrhage [18, 27]. Bronchial blockers and endoscopic balloon are suitable options for managing airway massive hemorrhage in the early stages or when there are definite bleeding sites and with a slow bleeding rate [25, 26]. Bronchial blockers need to be used in conjunction with endotracheal intubation, and endoscopic balloons need to be used under flexible electronic bronchoscope. Both devices require precise positioning and involve a relatively intricate procedure that may take longer to complete. Consequently, they are better suited for managing massive airway hemorrhage characterized by slow bleeding speed, such as venous or airway wall bleeding.

PVC endotracheal intubation for one-lung ventilation is not only simple and efficient, but also the most common and universal airway tool. The location of the intubation varies according to the bleeding site. When the bleeding is at the central tracheal airway beyond carina, intubate to the distal end of the bleeding site and use the cuff of the tube to compress the bleeding. When the bleeding site was in one lung lobe, the tip of endotracheal tube was inserted into the contralateral mainstem bronchus, and the cuff was fixed on the carina to prevent blood from overflowing from the affected side. In this way, the endotracheal tube played a dual role of one-lung ventilation and compression hemostasis. The positioning of the endotracheal tube is shown in Fig. 2. The flexible electronic bronchoscope can distinguish left mainstem bronchus, right mainstem bronchus, carina and other important anatomical parts in the endotracheal tube, and simultaneously judge the depth of the tube and the position of the cuff.

The average anatomical death space of the lung is about 150 ml. In the case of acute massive airway hemorrhage, bleeding lung should be blocked by the cuff in time by forming blood clot for hemostasis. The proper placement of the cuff on the carina can not only seal the blood extravasation from the bleeding lung, but also ensure the normal ventilation of the contralateral un-bleeding lung. At this time, intravenous hemostatic drugs are given to block the bleeding lung after the formation of a blood clot to stop the bleeding. Fully attract blood or secretions in the un-bleeding lung to ensure the ventilation function of un-bleeding lung is not affected. After 15 min of compression, the endotracheal intubation tube was then returned to the main airway and an electronic flexible bronchoscope was entered to examine the bleeding. If hemostasis is successful, thrombus debridement and follow-up treatment could be performed under jet ventilation again, trying to open up some lobes or segments of the lung. If the bleeding continued after thrombus removal, the current bronchoscopic interventional therapy should be abandoned, and lung isolation should be continued to wait for the opportunity for follow-up treatment, and if necessary, ECMO should be used to assist surgical hemostasis.

### Limitations

The overall sample size in this study was not very large due to the extremely low incidence of massive airway hemorrhage, especially the probability of bronchoscopy-associated acute massive airway hemorrhage. In addition, this study was a retrospective study, and some data could not be obtained only through medical records. And then, because of the urgency, severity, and lethality of massive airway hemorrhage, it would be unethical to conduct a randomized controlled trial to compare detailed differences between acute and non-acute massive airway hemorrhage. If the concept of acute massive airway hemorrhage is accepted, prospective studies can better observe the differences between the two hemorrhage concepts, their different effects on patients and the differences in treatment methods in subsequent cumulative and observational studies.

## Conclusion

Bronchoscopy-associated acute massive airway hemorrhage is defined as massive hemorrhage of more than 100 ml caused by bronchoscopy, rapid outflow of blood from the bleeding point, blurred vision of bronchoscopy, inability to stop bleeding quickly, and blood filling the alveoli, which seriously affects the oxygen supply function of the lung lobes. PVC single-lumen endotracheal intubation for lung isolation, with its characteristics of low difficulty, wide applicability and available in most hospitals, may reduce the intraoperative mortality of patients with bronchoscopy-associated acute massive airway hemorrhage.

## Data Availability

The datasets used and/or analyzed during the current study are available from the corresponding author.
